# The Workmen's Compensation Bill

**Published:** 1897-05-22

**Authors:** 


### THE WORKMEN'S COMPENSATION BILL.

Our readers will no doubt be interested to see how entirely the views we put forward, on the occasion of the introduction of the Workmen's Compensation Bill, as to the responsibility which will be thrown on the medical man by that measure, were endorsed during the discussion upon it in the House of Commons last Monday. Sir C. Dilke said a great deal of responsibility would rest upon the medical man in these cases. He would be the real arbitrator, and in many cases it would be his judgment, rather than that of the arbitrator, which would decide the case. The medical man, who had to decide whether a case was one of shamming, would have an immense responsibility thrown upon him. He thought it would be far better that the medical man should be appointed by the Home Office, and paid by the State than that he should be paid by the employer; and he hoped that the Government would give the House an assurance on that point such as would, he was sure, tend to make the Bill more popular, and secure better administration of it.

